# Host cell-derived lactate functions as an effector molecule in *Neisseria meningitidis* microcolony dispersal

**DOI:** 10.1371/journal.ppat.1006251

**Published:** 2017-04-06

**Authors:** Sara Sigurlásdóttir, Jakob Engman, Olaspers Sara Eriksson, Sunil D. Saroj, Nadezda Zguna, Pilar Lloris-Garcerá, Leopold L. Ilag, Ann-Beth Jonsson

**Affiliations:** 1 Department of Molecular Biosciences, The Wenner-Gren Institute, Stockholm University, Stockholm, Sweden; 2 Department of Environmental Science and Analytical Chemistry, Stockholm University, Stockholm, Sweden; University of Oxford, UNITED KINGDOM

## Abstract

The development of meningococcal disease, caused by the human pathogen *Neisseria meningitidis*, is preceded by the colonization of the epithelial layer in the nasopharynx. After initial adhesion to host cells meningococci form aggregates, through pilus-pilus interactions, termed microcolonies from which the bacteria later detach. Dispersal from microcolonies enables access to new colonization sites and facilitates the crossing of the cell barrier; however, this process is poorly understood. In this study, we used live-cell imaging to investigate the process of *N*. *meningitidis* microcolony dispersal. We show that direct contact with host cells is not required for microcolony dispersal, instead accumulation of a host-derived effector molecule induces microcolony dispersal. By using a host-cell free approach, we demonstrated that lactate, secreted from host cells, initiate rapid dispersal of microcolonies. Interestingly, metabolic utilization of lactate by the bacteria was not required for induction of dispersal, suggesting that lactate plays a role as a signaling molecule. Furthermore, *Neisseria gonorrhoeae* microcolony dispersal could also be induced by lactate. These findings reveal a role of host-secreted lactate in microcolony dispersal and virulence of pathogenic *Neisseria*.

## Author summary

The human restricted pathogen *Neisseria meningitidis* is a major cause of bacterial meningitis and sepsis worldwide. Colonization of the mucosal layer in the upper respiratory tract is essential to establish invasive disease. The initial interaction with host cells is characterized by bacterial proliferation and adhesion as aggregates, called microcolonies. Detachment from microcolonies in the nasopharyngeal epithelium facilitates crossing of the cell barrier that can result in invasive disease, yet this process is poorly understood. Here we demonstrate that lactate, an abundant molecule in host mucosal environments, induces *N*. *meningitidis* microcolony dispersal. Interestingly, metabolic utilization of lactate by the bacteria was not required for the process, suggesting that lactate play a role as a signaling molecule in pathogenic *Neisseria*. We propose that the microcolony dispersal in pathogenic *Neisseria* is influenced by environmental concentrations of lactate. These findings will assist in better understanding the transition from asymptomatic carriage to invasive disease.

## Introduction

Humans serve as the sole reservoir for the pathogen *Neisseria meningitidis*. The bacteria asymptomatically colonize the upper respiratory tract, with a prevalence of carriage ranging from 10 to 35% [1]. However, meningococci occasionally cross the epithelial mucosa and blood-brain barrier, causing life-threatening septicemia and meningitis [2]. Meningococcal adhesion to the epithelium in the nasopharynx is a prerequisite for colonization and pathogenicity [3], and this process can be divided into two steps. The initial interaction with cells is characterized by proliferation and adhesion as aggregates, called microcolonies. This stage is followed by the detachment of individual bacteria from the microcolonies, enabling relocation to new colonization sites or intimate adhesion to the cells in a single bacterial layer. Dispersal from microcolonies enables the meningococci to invade the mucosa and enter the circulation [4, 5].

The process of neisserial autoaggregation is highly dynamic and driven by interactions between type IV pili (Tfp). Tfp are one of the most multifaceted prokaryotic virulence factors mediating adhesion, autoaggregation, DNA uptake, biofilm formation, and twitching motility (reviewed in [6]). The dynamics of aggregation have been shown to be affected by several factors, including the minor pilin PilX, PilW and recently characterized polynucleotide phosphorylase (PNPase) [7–9]. The abundance of Tfp has also been shown to be critical for aggregation [10]. In addition to the amount of pili present on the surface of the bacterium, post-translational modifications of the major pilin subunit PilE have been shown to affect aggregation and dispersal. These modifications include O-linked glycosylation and addition of phosphocholine, phosphoethanolamine, or phosphoglycerol moieties [11–13]. The induction of PilE phosphoglycerol transferase B (PptB) and increased phosphoglycerol moieties on PilE favor the dissociation of pili bundles and subsequent microcolony dispersal [14]. A recent study showed that neisserial microcolony stability was affected by the oxygen concentration. Depletion of oxygen resulted in a loss of proton motive force (PMF), which affected the pilus-pilus interaction and led to a subsequent detachment of bacteria from microcolonies in liquid [15]. Although several factors have been shown to contribute to detachment of bacteria from microcolonies, the underlying mechanism is poorly understood.

In this study, we investigated the role of host cell-derived molecules in microcolony dispersal. Bacterial pathogens can sense host signaling molecules to acquire information about the host physiological status and alter virulence properties accordingly to adapt to distinct niches. In the gut, *Escherichia coli* O157:H7 (EHEC) sensing of ethanolamine, a source of carbon and nitrogen, and the sugar fucose can play an important role in regulating virulence genes involved in colonization. This mechanism occurs independently of metabolic utilization of the nutrients by the bacteria [16, 17]. Additionally, *Salmonella typhimurium* can utilize ethanolamine signaling to modulate metabolism and virulence [18]. *N*. *meningitidis* responds to a component present in human saliva, 4-hydroxyphenylacetic acid (4HPA), by altering gene regulation of the adhesins *nadA* and *mafA*, as well as metabolic pathways; 4HPA binds directly to and inhibits the activity of the FarR regulator [19–22]. This compound is a catabolite of aromatic amino acid metabolism, but it is unclear whether 4HPA is from the host cells or microbiota [23].

Here, we demonstrate that accumulation of a host cell-derived molecule, identified as lactate, is capable of inducing a rapid synchronized dispersal of meningococcal microcolonies. The dispersal was not dependent on the utilization of lactate by the bacterium. Additionally, the dispersal was independent of the previously reported mechanisms of microcolony dispersal mediated through depletion of the proton motive force. Altogether, our findings suggest that host-derived lactate plays an important role as a signaling molecule that mediates microcolony dispersal in pathogenic *Neisseria*.

## Results

### The dispersal of *N*. *meningitidis* microcolonies is synchronized and accelerated in the presence of live host cells

**Fig 1:**
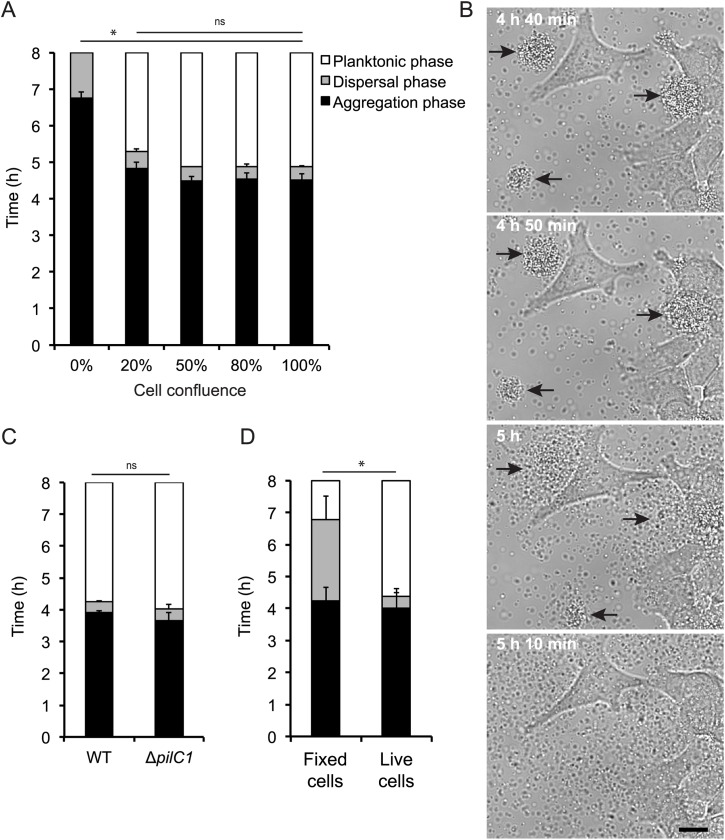
Host cells accelerate microcolony dispersal independent of direct contact. Microcolony formation and dispersal of *N*. *meningitidis* FAM20 (2 × 10^6^ CFU/ml) were monitored for 8 h by live-cell time-lapse microscopy after infection of FaDu cells. (A) FaDu cells at confluences of 100%, 80%, 50%, 20% and 0% were infected with FAM20. (B) The dispersal of FAM20 microcolonies on FaDu cells at 50% confluence was observed with live-cell time-lapse microscopy. Representative images are shown. Scale bar, 10 μm. (C) FaDu cells were infected with FAM20 or the Δ*pilC1* mutant. (D) FAM20 infecting fixed (3.7% paraformaldehyde) or live FaDu cells. Data represent the mean ± SD for at least three individual experiments. ^*^p < 0.05. ns, non-significant.

The aim of this study was to investigate the process of *N*. *meningitidis* microcolony dispersal. To evaluate the necessity of direct interaction with host cells, we monitored the timing of the dispersal upon infection of pharyngeal epithelial FaDu cells at different confluences using live-cell time-lapse microscopy. The dispersal phase began after approximately 4.5 h in the presence of cells and after 6.5 h in the absence of cells (Fig 1A). The total time the bacteria spent in the dispersal phase was drastically shorter in the presence of cells, lasting approximately 20 min, while in the absence of cells bacteria were still detaching from microcolonies after 8 h of incubation (Fig 1A). Microcolonies on cells at confluences of 100%, 80%, 50% and 20% showed no differences in dispersal phase length. Interestingly, the microcolonies that were not in direct contact with host cells (Fig 1B) also showed a synchronized and short dispersal phase similar to microcolonies that were in direct contact with host cells (S1A Fig).

To further examine the importance of direct contact prior to microcolony dispersal, we carried out the infection using an adhesion-deficient Δ*pilC1* mutant [24]. In an infection assay with FaDu cells, the Δ*pilC1* mutant showed a short and synchronized dispersal beginning after approximately 4 h of incubation, similar to that of the wild-type, supporting that cell contact is not required (Fig 1C). The adhesive properties of both the wild-type and the Δ*pilC1* mutant strains was confirmed by performing an adhesion assay. As expected, the Δ*pilC1* mutant adherence decreased by 10-fold in a comparison to the wild-type (S1B Fig).

The importance of live epithelial cells was further investigated by observing dispersal on FaDu cells that were fixed before infection. The microcolonies on fixed cells showed a long dispersal phase (Fig 1D). Microcolony dispersal was also monitored on the epithelial cell lines A549 (lung), Detroit 562 (pharynx), and Hec-1B (endometrium) to confirm that the observed effect of human cells was not specific to the FaDu cells. The length of the dispersal phase during the A549, Hec-1B and Detroit 562 infections was short, resembling that observed with the FaDu cells (S2A Fig).

Taken together, the data indicated that live host epithelial cells are important for *N*. *meningitidis* microcolony dispersal. The detachment of meningococci from microcolonies was synchronized and rapid in the presence of cells. The microcolony dispersal did, however, appear to be independent of direct contact between the bacteria and the cells.

### Host cell-conditioned medium induces a rapid and synchronized dispersal of the microcolonies

**Fig 2:**
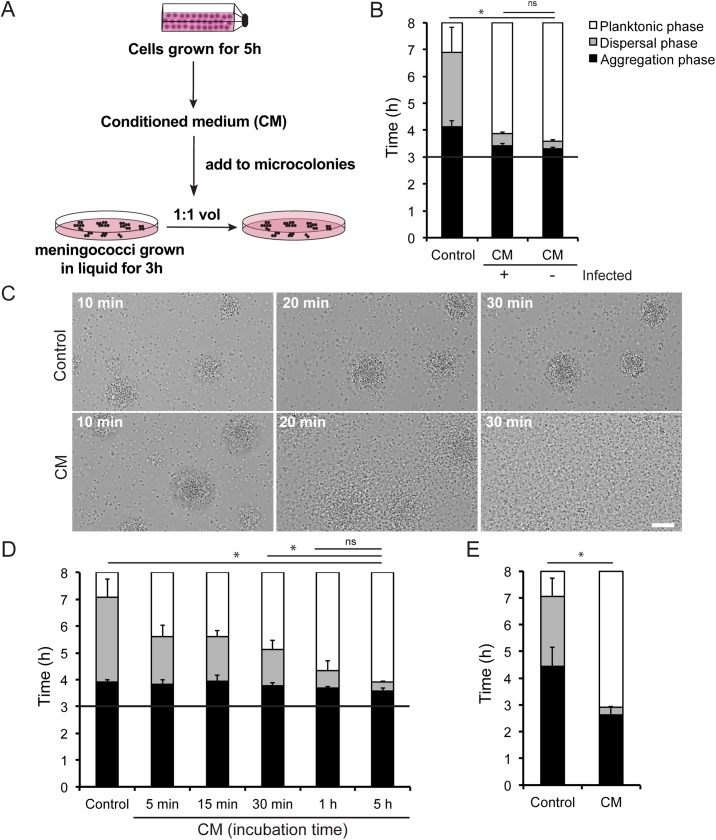
Cell-conditioned medium (CM) induces the dispersal of microcolonies. (A) Schematic drawing of the experimental set-up. Confluent FaDu cells were washed and incubated with DMEM. After 5 h, the CM was collected, sterile filtered, and added at a 1:1 volume ratio to bacterial meningococcal suspensions where microcolonies had been allowed to form for 3 h (initial bacterial concentration of 10^7^ CFU/ml). A black horizontal line in panels B and D represents the 3 h time point. Bacteria were observed by live-cell time-lapse microscopy. DMEM was used as a control. (B) Microcolony dispersal upon addition of CM from infected (+) or uninfected (-) cells to FAM20 bacteria in liquid. (C) Microcolony dispersal was examined by live-cell time-lapse microscopy after addition of CM. Representative images are shown 10, 20 and 30 min after induction. Scale bar, 10 μm. (D) Microcolony dispersal upon addition of CM collected from FaDu cells after 5, 15, and 30 min and 1 h and 5 h. (E) Microcolony dispersal of FAM20 resuspended (10^7^ CFU/ml) in either CM or DMEM (control) when the incubation was initiated. Data represent the mean ± SD of three independent experiments. ^*^p < 0.05. ns, non-significant.

Since direct contact with host cells was not necessary for microcolony dispersal, we chose to study whether a secreted compound(s) might stimulate this response. Since *N*. *meningitidis* in liquid without cells did not disperse as quickly, the compound(s) must originate from the host cells themselves or be generated during the cell-bacteria interaction. As bacteria had dispersed after 5 h in the presence of host cells, we collected medium after 5 h of incubation with both infected and uninfected FaDu cells. The conditioned medium (CM) from the infected or uninfected cells was added at a 1:1 volume ratio to bacterial suspensions in which microcolonies had been allowed to form for 3 h (Fig 2A). Regardless of whether the CM was from the infected or uninfected cells, a short dispersal phase was initiated after approximately 20 min and finished 20 min later (Fig 2B, 2C and S1 Movie). The microcolony dispersal of microcolonies, which had been treated with control medium, began after 1 h and was followed by a dispersal phase that lasted several hours (Fig 2B, 2C and S2 Movie). Induction assays performed with CM from other human cell lines induced dispersal similar to that of the CM from FaDu cells (S2B Fig).

We next investigated the time required for the medium to accumulate a sufficient amount of the microcolony dispersal factor(s). While CM collected between 5 min and 5 hours all accelerated the dispersal, the effect increased with time, with the fastest dispersal in CM collected after 5 h (Fig 2D). To determine whether the CM could also prevent microcolony formation, *N*. *meningitidis* were resuspended in CM at the beginning of the experiment. Interestingly, the bacteria formed microcolonies after approximately 2 h, similar to the bacteria in control medium, but dispersed 30 min later (Fig 2E). This indicates that *N*. *meningitidis* must first aggregate and can then respond to the CM.

**Fig 3:**
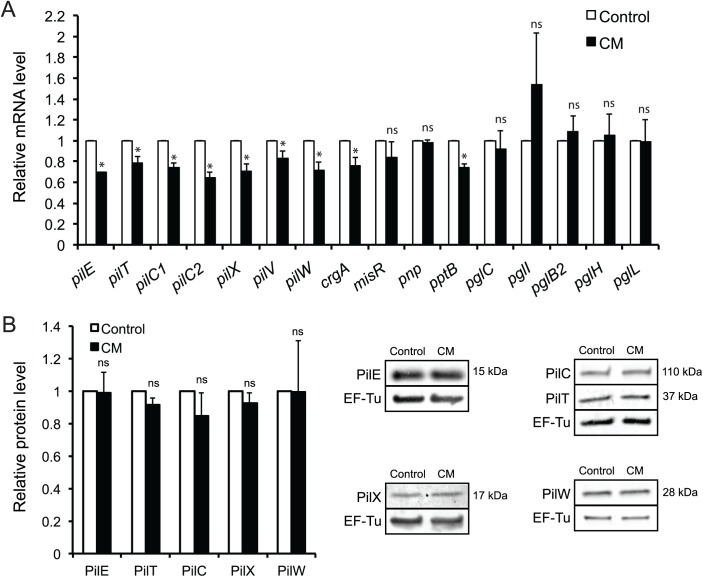
Expression of genes and proteins associated with *N*. *meningitidis* colonization upon addition of CM. The gene expression and protein level was examined upon addition of CM to preformed FAM20 microcolonies, with DMEM used as a control. (A) For quantitative real-time PCR, samples were taken 10 min after addition of CM. Target mRNA levels were normalized to the housekeeping gene coding for the 30S ribosomal protein RpsJ. (B) For Western blot analysis, samples were taken 10 min after addition of CM. After detection of the protein of interest, the membrane was stripped and the expression of EF-Tu was examined and used as a loading control. Gene and protein expression levels in the controls were set to a value of 1. Data represent the mean ± SD of three independent experiments. ^*^p < 0.05. ns, non-significant.

In response to CM, we detected significant changes in expression of the pilus-associated genes *pilE*, *pilT*, *pilC1*, *pilC2*, *pilX*, *pilV*, *pilW*, *pptB* [4, 7, 9, 14, 25, 26] and the transcriptional regulator *crgA* [27, 28], (Fig 3A). However, no significant changes were observed in expression of the transcriptional regulators *misR* or *pnp* [8, 29]. Additionally, no significant changes were observed in the genes *pglC*, *pglI*, *pglB2*, *pglH* and *pglL*, encoding for pilus posttranslational modification enzymes [30], (Fig 3A). Moreover, we analyzed the protein level of PilE, PilT, PilC, PilX and PilW at 10 min after addition of CM to microcolonies. We did not observe any significant changes on the protein level upon addition of CM (Fig 3B). The results indicate that expression of the proteins PilE, PilT, PilC, PilX and PilW, previously associated with meningococcal colonization, was not changed upon induction of microcolony dispersal. To summarize, these results indicate that *N*. *meningitidis* in microcolonies disperses in response to one or more compounds derived from host cells. The activity of the CM increased with incubation time, suggesting that the active molecule(s) accumulate over time.

### The host cell-derived inducer of rapid microcolony dispersal is a heat-stable, low-molecular weight compound(s)

**Fig 4:**
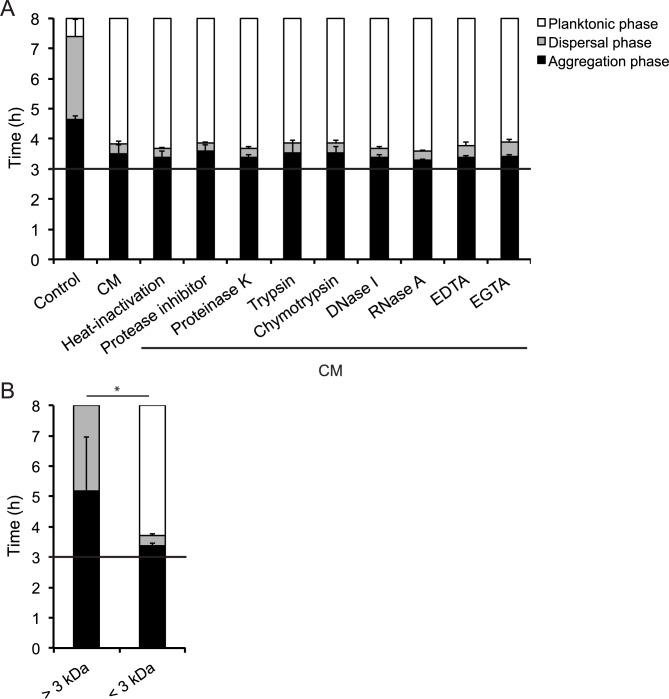
A heat-stable, low-molecular weight compound present in CM can induce microcolony dispersal. The CM was pretreated with 95°C heat, a protease inhibitor cocktail, proteinase K, trypsin, chymotrypsin, DNase I, RNase A, EDTA, or EGTA (A) or passaged through a 3 kDa cut-off filter (B) prior to induction assay. Microcolony dispersal was examined in live-cell time-lapse microscopy after addition of treated CM. A black horizontal line in panels represents the 3 h time point when treated CM was added to preformed microcolonies. Data represent the mean ± SD of three independent experiments. *p < 0.05.

To characterize the cell-derived compound(s) actively inducing meningococcal microcolony dispersal, we treated the CM with a protease inhibitor cocktail, proteinase K, trypsin, chymotrypsin, DNase I, RNase A, EDTA, or EGTA before addition to the microcolonies. The treated CM samples were fully active after the different treatments, as well as after heat inactivation (Fig 4A). These results indicated that the active component was not a protein, DNA, RNA, a divalent metal ion or a heat-sensitive molecule. Negative controls consisting of treated control medium did not affect microcolony dispersal (S3 Fig). In addition, the pH of the CM was measured and remained unchanged from the control medium. To further analyze the compound(s), we passed the CM over a 3 kDa cut-off filter. As shown in Fig 4B, the flow-through retained the ability to trigger microcolony dispersal, while the retentate, containing molecules larger than 3 kDa, did not, suggesting that the molecule is small in size. The treatments of the CM indicate that the compound(s) is smaller than 3 kDa, heat stable and is not a protein, a metal ion, DNA or RNA.

### Lactate is an effector molecule in the CM responsible for the accelerated dispersal

**Fig 5:**
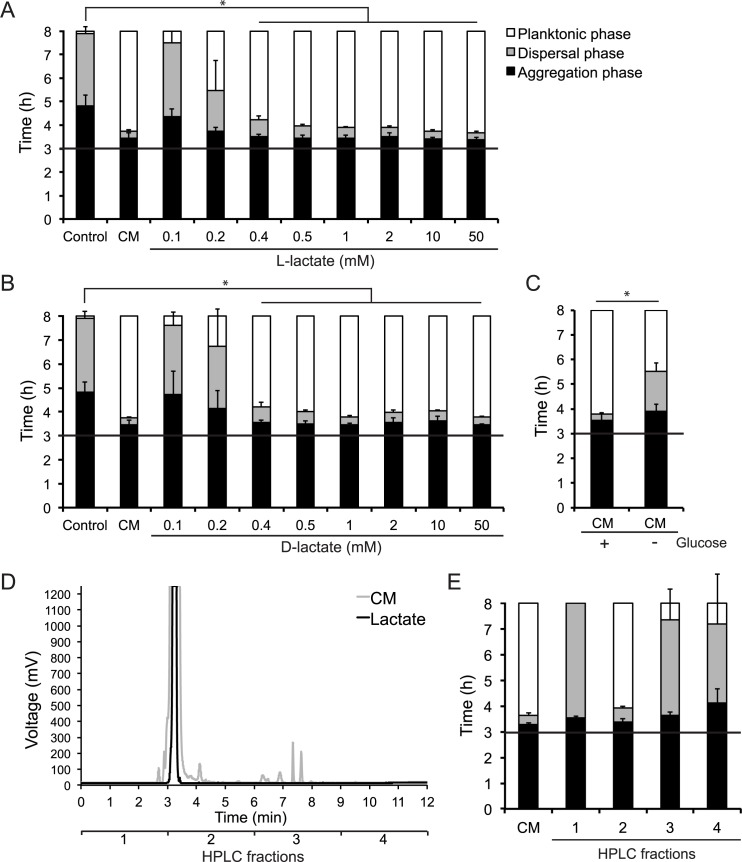
L-lactate and D-lactate induce microcolony dispersal. The timing of microcolony dispersal was examined in live-cell time-lapse microscopy after addition of L-lactate (A) and D-lactate (B), in final concentrations ranging from 0.1 mM– 50 mM, to preformed microcolonies. The same medium controls (DMEM and CM) were used for panel A and B since data was acquired from the same experiment. (C) CM, collected from host cells incubated in medium with (CM +) and without glucose (CM–), was added to microcolonies. For panel A-C, data represent the mean ± SD of three independent experiments. (D) Chromatograph of reversed-phase HPLC separation of CM (grey) and 50 mM D-lactate (black). Fractions were collected every three minutes for a total 12 min. For CM, one representative chromatogram out of two is shown. (E) HPLC fractions 1, 2, 3 and 4 collected from separation of CM were added to microcolonies. CM was used as a control. In panel E, one representative experiment out of two is shown, and data represent the mean ± SD of three images acquired per sample. A black horizontal line in panels represents the 3 h time point of induction. ^*^p < 0.05.

The accumulation of a low-molecular weight molecule over time in the CM led to the hypothesis that it may be a host cell metabolic end product. One such molecule is lactate, which is produced and excreted as a part of glucose fermentation in human cells. Lactate has previously been shown to stimulate metabolism and oxygen consumption in pathogenic *Neisseria* [31, 32]. A number of studies have examined a link between lactate metabolism and virulence in pathogenic *Neisseria* (reviewed in [33]). To test the ability of lactate to induce microcolony dispersal, we performed induction assays using L-lactate, which is the predominant lactate isoform found in the human body [34]. This assay showed that L-lactate was able to induce microcolony dispersal, similar to CM (Fig 5A). Interestingly, D-lactate was also able to induce dispersal in the same manner as L-lactate (Fig 5B). The minimum concentration required to induce dispersal was 0.4 mM for both L-lactate (Fig 5A) and D-lactate (Fig 5B). Since most lactate produced by human cells is derived from glucose fermentation, we collected CM from cells that were grown in absence of glucose. While the glucose depletion did not fully abolish the activity of the CM, it prolonged the dispersal phase by 1.5 hours compared to normal CM (Fig 5C).

Using reversed-phase HPLC, we separated the CM using an acetonitrile and water gradient. Fractions were collected every three minutes for a total 12 min (Fig 5D). The collected fractions were dried in a Speed-Vac and then resuspended in water, and the activity was examined in an induction assay. In addition, lactate was separated in the same way as CM. For both CM and lactate, fraction 2 was able to induce fast dispersal (Fig 5E and S4 Fig). The remaining fractions did not induce dispersal (Fig 5E and S4 Fig). The presence of lactate in the CM was confirmed by quantification. The concentration in the CM was 2 mM, and in the CM from cells grown in absence of glucose, the concentration was 0.15 mM (S5 Fig). This further supports the hypothesis that lactate is the effector molecule in the CM that induces rapid dispersal of microcolonies. To examine that the observed lactate-induced dispersion was not due to bactericidal effects exerted by lactate itself, we examined the viable count during the induction assays. We did not observe any difference in bacterial viability when DMEM containing 50 mM lactate was added to the microcolonies (S6 Fig).

**Fig 6:**
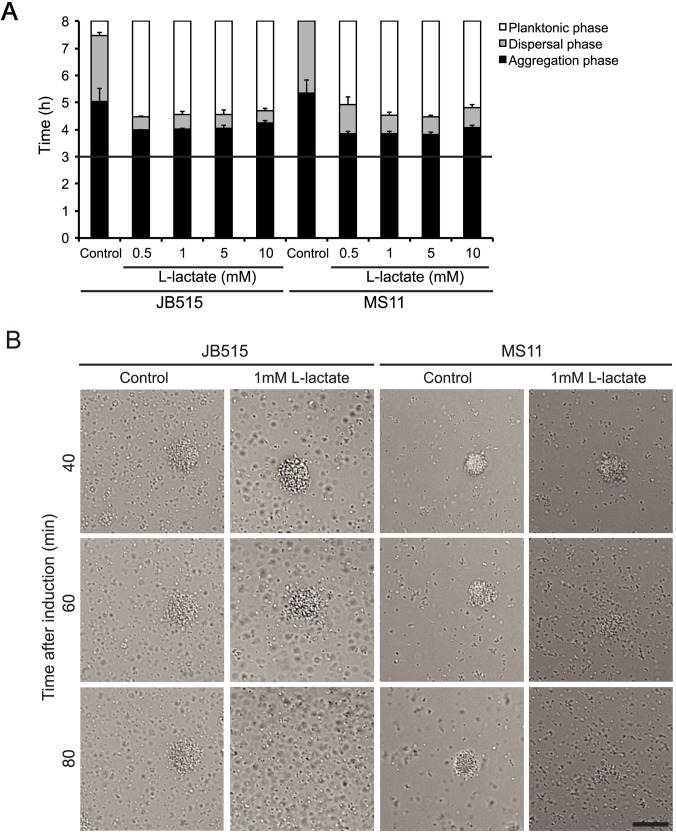
Lactate induces microcolony dispersal in *N*. *meningitidis* strain JB515 and *N*. *gonorrhoeae* strain MS11. (A) Bacteria (10^7^ CFU/ml) were allowed to form microcolonies for 3 h (black horizontal line) before addition of DMEM supplemented with lactate at final concentrations ranging from 0.5–10 mM. Microcolony dispersal was examined by live-cell microscopy for 8 h. Data represent the mean ± SD of three independent experiments. ^*^p < 0.05. (B) Representative images showing dispersal 40 min, 60 min and 80 min after addition of lactate (1 mM) to JB515 and MS11 microcolonies. Scale bar, 20 μm.

To establish that lactate-induced microcolony dispersal was not only restricted to the strain FAM20, we tested *N*. *meningitidis* serogroup W-135 strain JB515 and *N*. *gonorrrhoeae* strain MS11. Lactate induced similar effects on microcolony dispersal of *N*. *meningitidis* serogroup W strain JB515 and *Neisseria gonorrhoeae* strain MS11 (Fig 6).

Together, these data indicate an important role of lactate in microcolony dispersal of pathogenic *Neisseria*. Both L- and D-lactate at millimolar concentrations was sufficient to induce rapid dispersal.

### The metabolic utilization of lactate by *N*. *meningitidis* is not required for the induction of rapid microcolony dispersal

**Fig 7:**
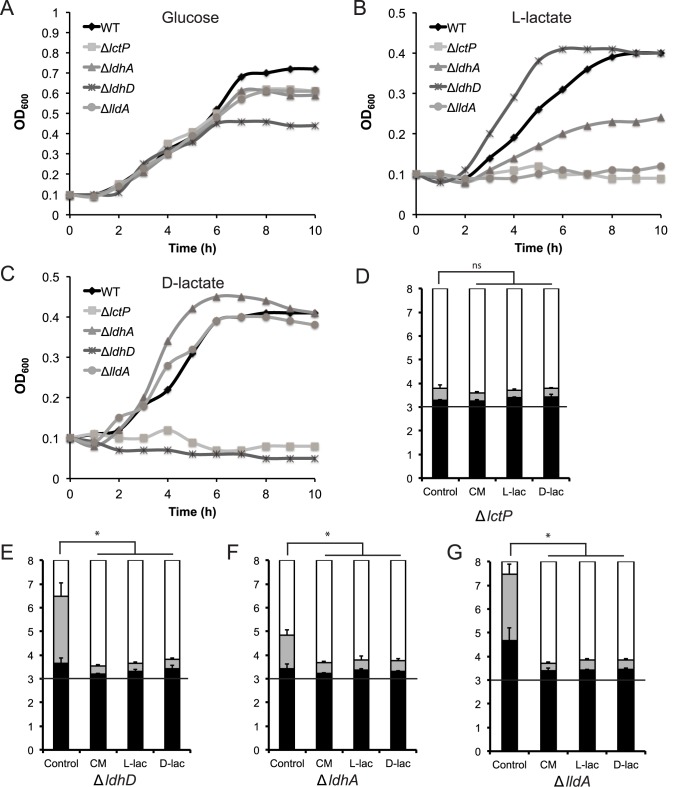
Lactate-induced microcolony dispersal is not dependent on metabolic utilization by *N*. *meningitidis*. Growth of FAM20 wild-type and its isogenic mutants Δ*lctP*, Δ*ldhA* Δ*ldhD*, Δ*lldA* in the presence of glucose (A), L-lactate (B) or D-lactate (C) as the major carbon source. (D-G) The timing of microcolony dispersal was examined after addition of DMEM (control), CM, L-lactate (10 mM) or D-lactate (10 mM) to preformed Δ*lctP* (D), Δ*ldhD* (E), Δ*ldhA* (F) or Δ*lldA* (G) microcolonies. Induction is represented by a black horizontal line. For panel A-C, one representative growth curve out of two is shown. For panel D-G, data represent the mean ± SD of three independent experiments. ^*^p < 0.05. ns, non-significant.

Meningococcal lactate metabolism is dependent on lactate permease (LctP) to take up lactate and a lactate dehydrogenase (LDH) to oxidize it to pyruvate [35, 36]. *N*. *meningitidis* is known to encode at least 3 LDHs. Two are membrane-bound respiratory LDHs, specific for either L-lactate (*lldA*) or D-lactate (*ldhD*), and one is a cytoplasmic D-lactate LDH (*ldhA*) [37–39]. To further assess the importance of lactate in microcolony dispersal, we created *ldhD*, *lldA*, *ldhA* and *lctP* deletion mutants of FAM20. Growth analysis was performed to determine the ability of the strains to grow on lactate as a carbon source (Fig 7A–7C). Consistent with previous studies, the Δ*lctP* mutant was unable to utilize either L- or D-lactate but grew normally in the presence of glucose. The Δ*ldhD* mutant was unable to utilize D-lactate but grew normally on both L-lactate and glucose. With L-lactate as a carbon source, the Δ*lldA* mutant was unable to grow and the Δ*ldhA* mutant was impaired in growth (Fig 7A–7C).

To investigate whether lactate uptake and metabolism were required for induction of dispersal, Δ*lctP*, Δ*ldhA*, Δ*lldA* and Δ*ldhD* mutants were used in induction assays. Interestingly, deletion of *lctP* accelerated microcolony dispersal in control medium (Fig 7D). Although Δ*ldhD* mutant was unable to utilize D-lactate, microcolony dispersal could be induced by addition of D-lactate (Fig 7E). The same could be observed by addition of L-lactate to microcolonies formed by Δ*ldhA* and Δ*lldA* mutants (Fig 7F–7G). This suggests that lactate does not need to be metabolized to induce rapid dispersal.

### Lactate-induced dispersal of microcolonies is independent of oxygen depletion

**Fig 8:**
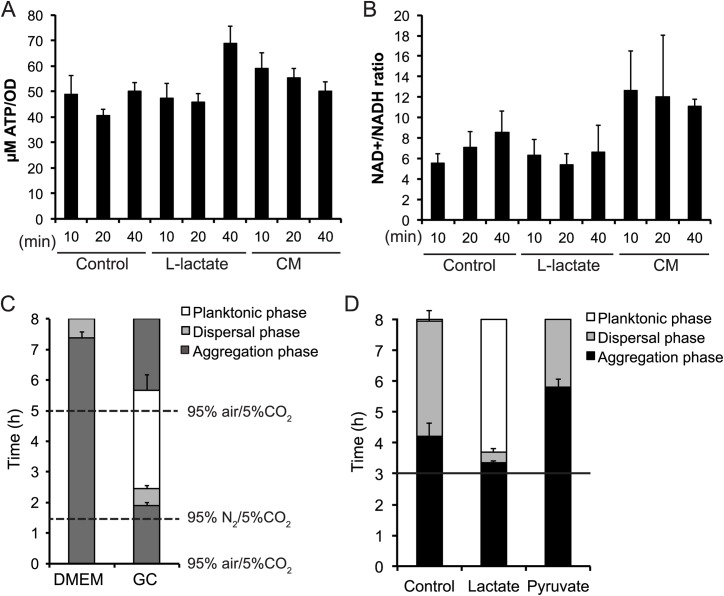
Lactate- and CM-induced microcolony dispersal of *N*. *meningitidis* occurs independent of oxygen depletion. Samples were taken 10, 20 and 40 min after addition of DMEM (control), CM or L-lactate (10 mM) to FAM20 microcolonies. The concentration of ATP (A) and the NAD^+^/NADH (B) ratio were measured. (C) Microcolony stability in both DMEM and GC liquid during oxygen depletion. At 1.5 h the oxygen level was set to 0% by a 95% N_2_/5% CO_2_ flow. At 5 h, the oxygen level was reversed to 95% air/5% CO_2_. The dotted lines in the panel represent the 1.5 h and 5 h time points. (D) Addition of DMEM (control), L-lactate (10 mM) and pyruvate (10 mM) to preformed microcolonies. Data represents the mean ± SD of three independent experiments. A black horizontal line in panel D represents the 3 h time point.

One factor that controls microcolony dispersal in *N*. *gonorrhoeae* is the oxygen concentration. Oxygen depletion leads to dispersal through pili retraction, which is mediated by depletion of the proton motive force (PMF) [15]. Since it has been shown that lactate stimulates growth and oxygen consumption in the presence of glucose [32], one hypothesis was that this increased oxygen consumption leads to oxygen depletion and dispersal. Changes in PMF affect the ATP level and the NAD^+^/NADH ratio. To determine if depletion of the PMF caused dispersal in response to lactate addition, we measured the concentration of ATP and the ratio of NAD^+^/NADH after induction of microcolony dispersal with either lactate or CM. Our results showed that the ATP concentration and the NAD^+^/NADH ratio remained similar or increased slightly (Fig 8A and 8B). The addition of carbonyl cyanide *m*-chlorophenyl hydrazone (CCCP, 25 μM) caused a decrease in ATP concentration (S7 Fig). This suggests that a depletion of the PMF caused by oxygen depletion was not the cause of dispersal. In addition, we were unable to induce dispersal by decreasing the environmental oxygen concentration of bacteria grown in DMEM (Fig 8C), in contrast to bacteria grown in GC as shown by Dewenter *et al*. [15]. However, one of the reasons for the observed differences in GC and DMEM for the role of oxygen depletion in microcolony dispersal may be due to differences in the rate at which oxygen depletion takes places in both the medium. Further investigation is required to conclusively determine the role of oxygen depletion in the lactate-induced accelerated microcolony dispersal.

Induction assays were also performed with addition of pyruvate since it is the metabolite directly downstream of lactate and has been shown to have the same stimulatory effect on *Neisseria* metabolism as lactate [40]. Addition of pyruvate did not induce microcolony dispersal, which further suggests that the effect is not due to an increase in metabolic rate (Fig 8D).

These data indicate that the observed effect on dispersal is independent of the previously described mechanism that relied on depletion of oxygen and the membrane potential.

## Discussion

**Fig 9:**
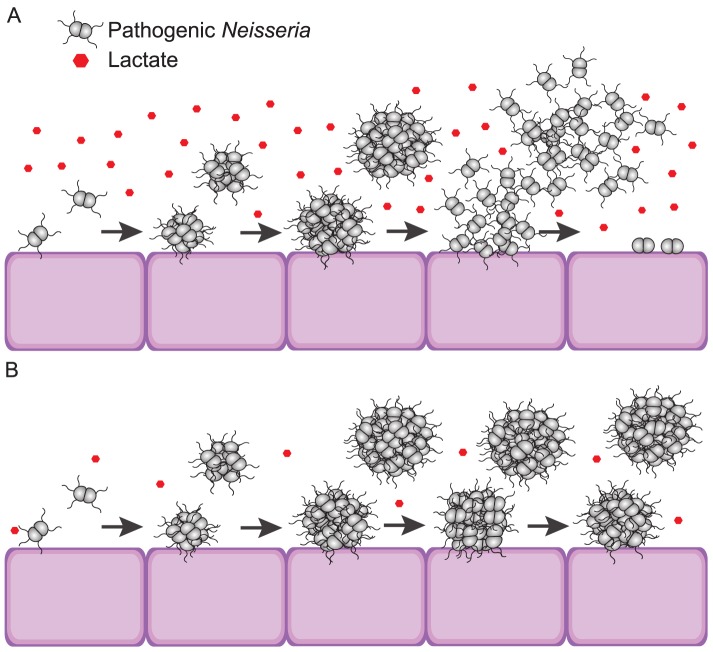
Environmental concentration of lactate influences microcolony dispersal in pathogenic *Neisseria*. Proposed model of lactate-induced microcolony dispersal in pathogenic *Neisseria*. (A) In presence of high lactate concentration the microcolony dispersal of pathogenic *Neisseria* is rapid regardless of whether microcolonies are in direct contact with host cells or not. (B) In low lactate concentrations, pathogenic *Neisseria* remain in microcolonies.

The transition from nasopharyngeal colonization to an invasive infection is a crucial step in meningococcal pathogenicity. The detachment of meningococci from microcolonies allows bacteria to colonize new sites and to act as single cells that can cross the epithelial barrier [5, 14]. In this study, we investigated the importance of epithelial cells and cell-derived factors for microcolony dispersal. We demonstrated that the previously observed short and synchronized dispersal of microcolonies [41] requires the presence of live epithelial cells but not direct contact between cells and bacteria. Microcolony dispersal could be induced by a low-molecular weight host cell-derived factor that accumulated in cell-conditioned medium (i.e., CM) in absence of infectious agent. Furthermore, we showed that lactate is the active inducer of rapid microcolony dispersal in both *N*. *meningitidis* and *N*. *gonorrhoeae*. We propose that the microcolony dispersal in pathogenic *Neisseria* is influenced by environmental concentrations of lactate (Fig 9). Our data reveal a potential role of lactate as an effector molecule in colonization of pathogenic *Neisseria*.

To survive and proliferate within the host, bacteria must be able to sense and respond to changes in the environment. Many pathogens are known to respond to nutrient availability in the environment to regulate virulence-related gene expression (reviewed in [42]). Several studies have highlighted the importance of energy metabolism for meningococcal colonization [35, 43]. *Neisseria* is restricted to glucose, pyruvate and lactate as carbon sources [36]. While the concentrations of pyruvate generally are low, glucose and/or lactate are present in the tissues colonized by *Neisseria*. During normal conditions, L-lactate is the dominant isoform found, but D-lactate can also be present as a result of fermentation from bacteria such as lactobacilli [44]. Lactate is the major carbon source on mucosal surfaces where lactic acid bacteria colonize, and the concentration of lactate in nasopharyngeal tissue can be higher than 1 mM [35]. Lactate is also present at high concentrations in the female genital tract colonized by *N*. *gonorrhoeae*, where it can go up to more than 6 mM. In the bloodstream and cerebrospinal fluid, glucose serves as the major carbon source, although the lactate concentration can be higher than 1 mM [36]. Lactate and pyruvate predominate as carbon sources within phagocytes [45]. Our results showed that both lactate isoforms were able to induce microcolony dispersal at 0.4 mM. This is within the range of physiological concentrations encountered by pathogenic *Neisseria in vivo* [35, 36]. As pathogenic *Neisseria* metabolize and grow faster in the presence of lactate, receiving nutritional signals from the environment during favorable growth conditions and detaching from microcolonies could be a survival strategy.

Our results showed that lactate induces microcolony dispersal independent of the presence of the bacterial LDH that oxidizes it. These results show that the observed effect is not due to the previously characterized burst in respiration and growth after lactate addition in the presence of glucose [32]. This is also supported by the observation that pyruvate did not induce dispersal but promoted growth in a similar manner to lactate. These results indicate that the previously described mechanism of neisserial microcolony dispersal triggered by oxygen depletion and subsequent membrane depolarization is not the mechanism in this case [15]. To test this, we measured the concentration of ATP and the ratio of NAD^+^/NADH during dispersal. We did not observe any decrease in either the ATP concentration or the NAD^+^/NADH ratio in the dispersed bacteria, suggesting that no dramatic decrease in the PMF occurred during the process.

In the absence of lactate the dispersal phase lasted for several hours. However, when the gene encoding for the lactate permease (*lctP*), required for lactate uptake, was deleted we observed accelerated dispersal even in absence of lactate. This result suggests a role for LctP as a negative regulator of microcolony dispersal since deletion of the *lctP* resulted in microcolony dispersal. It is worthwhile to mention that lactate permease has been shown to function in a PMF-dependent manner in bacteria [46, 47]. Disruption of the PMF may therefore affect microcolony dispersal, as shown by Dewenter *et al* [15], indirectly by a change in lactate permease activity. However, there is also a possibility that the absence of LctP can affect lactate export and lead to accumulation of intracellular lactate produced by the bacterium and cause dispersal.

Deletion of *lctP* in *N*. *meningitidis* has previously been shown to attenuate nasopharyngeal colonization despite for increased initial adherence to host cells. It has also been shown to affect the ability to cause bloodstream infection and cause an increase in sensitivity to complement-mediated killing [31, 35]. The incompetence to acquire and utilize nutrients, like lactate, important for growth plays a crucial role in colonization. Since microcolony dispersal seems to be a highly regulated in pathogenic *Neisseria*, deregulation of the process like we have observed with the Δ*lctP* mutant, could also affect the ability of the bacteria to colonize in efficient way.

Although lactate is able to induce microcolony dispersal, we cannot exclude the possibility that other host derived factors present in the CM and *in vivo* might have the same stimulatory effect as lactate. However, collection of CM from host cells grown in absence of glucose, and thus inhibited in their ability to produce and secrete lactate, significantly reduced the stimulatory activity.

Earlier studies have shown that meningococci bound to epithelial cells modulate their gene expression, enabling them to form intimate contacts with the cells [14, 27–29, 48]. However, we did not detect any upregulation of the transcription regulator *misR* or the MisR/S-regulated genes *pptB*, *pilC1* and *crgA* after inducing dispersal with CM. One possible explanation is that meningococci respond differently when interacting with cells than when they are treated with CM. However, our results from the infection assays using different cell confluences and the adhesion-deficient Δ*pilC1* mutant showed that direct contact with cells is not essential for the timing of the dispersal phase or for its short time span. Additionally, our qPCR analysis included genes important for meningococcal piliation and pilus aggregative properties (*pilC1*, *pilC2*, *pilE*, *pilT*, *pilX*, *pilV* and *pilW*) [4, 7, 9, 10]. We did observe significant changes in their mRNA levels upon induction with CM. However, when we analyzed the protein level of PilE, PilT, PilC, PilX and PilW we did not detect any changes.

Post-translational modifications of the PilE subunit can also modulate Tfp function. Tfp of *Neisseria* can be further grouped into class I and class II pili. In majority of our study we used the meningococcal C strain FAM20 that is known to express class II pili. Recent findings by Gault *et al* indicate that class II pili can contain up to 5 glycosylation sites and deletions of glycosylation genes in these strains affect adhesion, aggregation and successful pilus assembly. The multiple glycosylation sites are suggested as a way to avoid immune recognition as class II pilus expressing strains lack the ability to vary the antigenicity of the pilus fiber [30]. By using two additional strains, meningococcal W-135 strain JB515 and *N*. *gonorrhoeae* MS11, we observed that lactate induced dispersion occurs in both class I and class II pilus expressing strains. Although no upregulation was observed in *pptB* expression and no changes were observed in the expression of genes involved in glycosylation modifications we cannot exclude that post-translational modifications play a role in response to CM and lactate stimulation. Moreover, PilE can also be modulated by phosphocholine and phosphoethanolamine [49]. The absence of these modifications has been shown to affect pilus bundling but no changes in functionality have been observed [50].

In conclusion, the work presented here demonstrates that lactate, secreted from host cells, can stimulate the dispersal of microcolonies in pathogenic *Neisseria*. The metabolic utilization of lactate by *N*. *meningitidis* was not required for induction of rapid dispersal. In the future, it would be interesting to identify adhesive and general properties of dispersed bacteria upon interaction with lactate. This study provides a basis for future research to further investigate the role of lactate as a signaling molecule influencing disease progression in pathogenic *Neisseria*.

## Materials and methods

### Bacterial strains and growth conditions

The *Neisseria meningitidis* FAM20 serogroup C strain and mutant deficient in PilC1 have been described previously [24, 51]. The *Neisseria meningitidis* JB515 serogroup W strain and *Neisseria gonorrhoeae* MS11 strain have been described previously [24, 52]. The strains were grown on GC agar (Acumedia) supplemented with 1% Kelloggs’ for 18 h at 37°C in a 5% CO_2_ environment. Antibiotics for selection of FAM20 mutant strains were used in following concentrations: tetracycline 1 μg/ml, kanamycin 50 μg/ml and spectinomycin 40 μg/ml.

### Cell lines and growth conditions

The human epithelial cell lines FaDu (ATCC HTB-43), A549 (ATCC CCL-185), and Detroit 562 (ATCC CCL-138) and the human endometrial epithelial cell line Hec-1B (ATCC HTB-113) were maintained in Dulbecco’s modified Eagle’s medium containing GlutaMAX and pyruvate (DMEM; Thermofisher) and supplemented with 10% heat inactivated fetal bovine serum (FBS; Sigma-Aldrich). For live-cell imaging, the cells were grown to 100% confluence unless stated otherwise. Prior to the experiments, the cells were washed and the medium exchanged to fresh DMEM without FBS. For fixation, the FaDu cells were treated with 3.7% paraformaldehyde (Sigma-Aldrich) for 10 min and then washed extensively.

### Collagen coating of cell culture plates

For live-cell imaging, the cells were seeded in 24-well poly-D-lysine-coated glass-bottom plates (MatTek) precoated with collagen type 1 from calf skin (Sigma-Aldrich). Wells were coated with 0.5 ml of a 0.01% collagen solution for 1 h at room temperature and then washed with PBS and water. The plates were dried under laminar airflow for 30–60 min and stored at 4°C until they were seeded with cells.

### Time-lapse microscopy

*N*. *meningitidis* FAM20 and its isogenic mutants were resuspended (2 × 10^6^ CFU/ml) in prewarmed medium, filtered through a 5-μm pore filter to break apart preexisting bacterial aggregates, and used to infect cells at confluences of 20%, 50%, 80% and 100% (MOI of 10 for 100% confluent cell layer) and 0% (absence of cells) in 24-well collagen coated poly-D-lysine-coated glass-bottom plates (MatTek). Infection assays were performed in DMEM without FBS, and the bacteria were gently centrifuged onto the cell surface (200 × *g*; 5 min). When observing aggregation in liquid during induction assays, bacteria were resuspended in prewarmed DMEM (10^7^ CFU/ml) containing 1% FBS, filtered through a 5-μm pore filter and added to 24-well glass-bottom plates (MatTek). While FBS was included in the medium for the assays without cells to facilitate the formation of microcolonies, it was excluded from the assays with cells to facilitate the later purification of soluble compounds triggering microcolony dispersal.

The bacteria were observed under a live-cell microscope (Axiovert Z1, Zeiss) at 37°C in a 5% CO_2_ environment. Three images per well were acquired every 5–10 min for 8 h using a 40× objective. The aggregation phase was defined as the time from the start of incubation to the start of microcolony dispersal. The dispersal phase was defined as the time from when the bacteria in the microcolonies began to spread out until microcolonies were no longer present. The planktonic phase was defined as the time when all bacteria had dispersed from the microcolonies and were present as single cells.

### Adhesion assays

*N*. *meningitidis* FAM20 and Δ*pilC1* were resuspended (2 × 10^6^ CFU/ml) in prewarmed medium, filtered through a 5-μm pore filter to break apart preexisting bacterial aggregates, and used to infect FaDu cells at 100% confluence at MOI of 10 in 24-well plates. The bacteria were gently centrifuged onto the cell surface (200 × *g*; 5 min) and incubated for 4 h at 37°C in a 5% CO_2_ environment. After incubation, unbound bacteria were removed by washing 3 times. The adherence was estimated from the viable counts by lysing the FaDu cells with 1% saponin in DMEM for 10 min and plating serial dilutions on GC agar.

### Collection and treatment of conditioned medium

Cells were grown in either 75 cm^2^ or 25 cm^2^ cell culture flasks to 90–100% confluence, washed once with PBS, and, unless stated otherwise, incubated in fresh DMEM (5 ml per 25 cm^2^) for 5 h to obtain conditioned medium (CM). CM was collected from uninfected cells unless stated otherwise. For the CM from infected cells, FaDu cells were infected with FAM20 at an MOI of 10 as described above for 5 h. The CM obtained were sterile-filtered and used in the induction assays. For the analysis of the active compound(s) in the CM from uninfected cells, the following treatments were applied: (a) heat inactivation by incubation at 95°C for 5 min, followed by sterile filtration to remove precipitates; (b) protease inhibitor cocktail (1:100 final dilution; P1860, Sigma-Aldrich), added to the CM 10 min before induction; (c) proteinase K (100 μg/ml; P2308, Sigma-Aldrich) added to CM, incubated overnight at 56°C and inactivated at 95°C for 20 min before sterile filtration to remove precipitates; and (d) trypsin (200 μg/ml; T1426, Sigma-Aldrich) in CM for 1 h at 37°C or chymotrypsin (200 μg/ml; C3142, Sigma-Aldrich) in CM for 1 h at 30°C, followed by inactivation with the protease inhibitor cocktail (1:100) for 10 min before induction. For the DNA and RNA digestions, final concentrations of 10 U/ml DNase I (D5025, Sigma-Aldrich) or 10 μg/ml RNase A (R4875, Sigma-Aldrich) were incubated with the CM for 2 h at 37°C. EDTA (E5134, Sigma-Aldrich) and EGTA (E4378, Sigma-Aldrich) were used as metal ion chelators at final concentrations of 2 mM. As negative controls, the same treatments were performed on DMEM and used in the induction assays. The CM was also filtered through a 3 kDa cut-off Amicon Ultra centrifugal filter device (Millipore).

### Induction assay

*N*. *meningitidis* strain FAM20 and its isogenic mutants, *N*. *meningitidis* strain JB515 or *N*. *gonorrhoeae* strain MS11 were resuspended (10^7^ CFU/ml) in prewarmed medium containing 1% FBS and filtered through a 5-μm pore filter to break apart preexisting bacterial aggregates. The bacteria were grown in 24-well glass-bottom plates (MatTek) in either 0.5 ml (wild-type compared to isogenic mutants) or 1 ml and allowed to form microcolonies for 3 h in the live-cell microscope at 37°C in a 5% CO_2_ environment. Prewarmed DMEM, CM, DMEM supplemented with pyruvate (final concentration 10 mM, sodium pyruvate, 11360070, Thermo Fisher scientific) or lactate (sodium D-lactate; L7022, sodium D-lactate; 71716, Sigma Aldrich) at the indicated concentrations were then gently added to the microcolonies at a 1:1 volume ratio. The 3 h time point was chosen because the microcolonies had formed, but at least 1 h remained until they would spontaneously begin to disperse. Three images per well were acquired every 5–10 min for 8 h using a 20x or 40× objective.

### Quantitative real-time PCR analysis

Induction assays with CM from non-infected cells and control DMEM were performed on FAM20 wild-type as indicated above in 12-well glass-bottom plates in 2 ml. Samples were collected 10 min after addition of CM or DMEM and resuspended in twice the volume of RNAprotect Bacteria Reagent (Qiagen). The sample:RNAprotect mixtures were vortexed at high speed (5 s), incubated for 5 min at room temperature, pelleted by centrifugation for 15 min at 4000 × *g* and purified using the RNeasy plus mini kit (Qiagen) according to the manufacturer’s protocol. The RNA yield and purity were assessed using a NanoDrop 8000, and reverse transcription was performed with random hexamers using a SuperScript VILO Master Mix (Thermofisher). The resulting cDNA was amplified using LightCycler 480 SYBR Green I Master mix (Roche) in a LightCycler 480 Real-Time PCR System. The PCR program was adapted from the manufacturer using an annealing temperature of 55°C or 60°C. The 30S ribosomal protein *rpsJ* was used as a reference gene. Table 1 lists the primers used in the assay. Primers were used at a final concentration of 500 nM except the primer pairs for *pilT* and *pilC2*, which were diluted to 250 nM. The analysis was performed with the LightCycler 480 Real-Time PCR System software using the comparative cycle threshold method. The target mRNA levels in the samples were normalized to the reference gene and then compared to the value of the non-induced DMEM control sample. Primer-pair specificity was controlled for by analyzing the melting curves.

**Table 1:** Primers used in this study.

Primer	Sequence (5′–3′)	Reference
rpsJ_fwd	TTGGAAATCCGCACCCACTT	[54]
rpsJ_rev	TACATCAACACCGGCCGACAAA	[54]
pilE_fwd	TATTCCGACAACGGCACATTCCC	[54]
pilE_rev	CCTTCAACCTTAACCGATGCCA	[54]
pilT_fwd	GTCGACCGTATCGTGGACGTATT	[55]
pilT_rev	TTCAGCAGGTTTTGGGAGATGAC	[55]
pilC1_fwd	CAATGCCCCCAACTTTTCTA	This work
pilC1_rev	AATAGGGTGGTCTCGTGTCG	This work
pilC2_fwd	TATAGCCATCAGGACGCACA	This work
pilC2_rev	ACCGAACCTACCAAGCTCCT	This work
pilX_fwd	CGGGGACGGGTTATACTTT	This work
pilX_rev	GGCATCACGGCATTTGTATC	This work
pilV_fwd	GGCAGCCATCCATTACG	This work
pilV_rev	GCCGAATCATTTACCCACAA	This work
pilW_fwd	AACTACGGCTGGTTCCTGTG	[8]
pilW_rev	GTGCGCGCCAGTTCTTTAAA	[8]
pptB_fwd	AAGGCGTGGAAGTCATCATC	This work
pptB_rev	TGTTTGAGGTAGGTAGCGGAAGGT	This work
misR_fwd	CAACCTGCTCGAAGTCCTTT	This work
misR_rev	GATGCTGGAGATGGTGTACGTC	This work
pnp_fwd	TTGGGCGACGAAGACCACTT	[8]
pnp_rev	TGTGCAGACGCGCTTCTTTG	[8]
crgA_fwd	CTCGTTGTGCCTTTCAGGTT	This work
crgA_rev	TTAACTTTCCTTCAGCGATGTC	This work
pglC_fw	GCGATTATCGTGGTTCACCT	This work
pglC_rev	ACCGGTGGTCATGATTTTGT	This work
pglI_fw	GAAGAACACCTGCCCCTGTA	This work
pglI_rev	CCATCAGGTAAACGGCTTGT	This work
pglB2_fw	ATTTTCAATCTGGCGGTACG	This work
pglB2_rev	AAATTTCGGGTGAGCGTATG	This work
pglH_fw	TGCAGTCGGTTACCAACAAA	This work
pglH_rev	TTATCGGCTTGAACGAAACC	This work
pglL_fw	GGCCTGATTGTCCTGTTGTT	This work
pglL_rev	GGGTAACGATGCGTTCTTGT	This work
ldhA_up_fw	ATGCCGTCTGAATTCAGTGTATTATGCCGT	This work
ldhA_up_rev	TATCGTATGGGGCTGGTTTTCACGCCAATTTGCG	This work
ldhA_tet_fw	GCGTGAAAACCAGCCCCATACGATATAAGTTG	This work
ldhA_tet_rev	TCATCACGCCGTTTCCATTCAGGTCGAGGTG	This work
ldhA_dn_fw	GACCTGAATGGAAACGGCGTGATGATTATCAACA	This work
ldhA_dn_rev	TAAAACACGTCAGCCGTCG	This work
ldhD_up_fw	CGTTATAATGCCGTTTCTCTG	This work
ldhD_up_rev	GGTCACTAATACGTATTGTTCGGTTTTCGC	This work
ldhD_kan_fw	CCGAACAATACGTATTAGTGACCTGTAGAATTCGAG	This work
ldhD_kan_rev	TTGGTCGGGTCTTAGAAAAACTCATCGAGCATCAAATG	This work
ldhD_dn_fw	AGTTTTTCTAAGACCCGACCAACAGCTTCA	This work
ldhD_dn_rev	TTAAACCAGTACGGCGTTACC	This work
lldA_up_fw	ATTGCGCGATTGGCTGTTTA	This work
lldA_up_rev	ACCCTAGAGCACGAACCCGAATCGATGTAA	This work
lldA_spc_fw	TCGGGTTCGTGCTCTAGGGTCCCCAATTAATTAGT	This work
lldA_spc_rev	ACGCCTTCAAAGTAAAGCCCTCGCTAGAT	This work
lldA_dn_fw	GCTTTACTTTGAAGGCGTGGGCTTTGG	This work
lldA_dn_rev	GCCAAATTTTAATGCCGTCC	This work
lctP_up_fw	ATGCCGTCTGAAGGGCGTTATCGATAAAATGCT	This work
lctP_up_rev	TATCGTATGGGGCTGAGTTTGATGGCGTAAATCAGC	This work
lctP_tet_fw	CCATCAAACTCAGCCCCATACGATATAAGTTG	This work
lctP_tet_rev	TTGGACACGGTTTCCATTCAGGTCGAGGTG	This work
lctP_dn_fw	GACCTGAATGGAAACCGTGTCCAACCTGACTTTC	This work
lctP_dn_rev	TGCTGCCGCTATGGATGGT	This work

### Western blot analysis

Induction assays with CM from noninfected cells were performed on FAM20 wild-type as indicated above in 12-well in glass-bottom plates (MatTek) using DMEM as a negative control. Samples were collected after 10 min, centrifugated and resuspended in 1x sample buffer containing β-mercaptoethanol. The samples were heated for 5 min at 95°C and separated on a 4–15% gradient SDS-PAGE gels (Bio-Rad). After a transfer to Immobilon-P membrane (Millipore) rabbit polyclonal PilE (1:5000), PilT (1:10000), PilC (1:1000), PilX (1:1250) and PilW (1:2000) antibodies were used to detect protein expression levels [7, 9, 51, 53]. After detection the membrane was stripped and monoclonal mouse antibody (Hycult Biotech) diluted 1:2000 was used to detect EF-Tu elongation factor as a loading control. Infrared (IR)- reactive dye conjugated goat anti-mouse or goat anti-rabbit antibodies (Li-Cor) were used as a secondary antibodies. Membranes were examined in Odyssey IR scanner at standardized 700 and 800nm. Image J analysis software (version 1.48) was used for quantification of band intensity.

### Fractionation of CM and lactate

Fractionation of CM and D-lactate (50 mM) was performed using a preparative liquid chromatography (LC) method. A Varian Prostar 230 HPLC pump (Walnut Creek, USA) delivered the binary mobile phase (MP) at the flow rate of 4.0 mL/min according to the programmed gradient (MP A: water and MP B: acetonitrile) as follows: 0.0 min, 2% B; 2.0 min, 2% B; 10.0 min, 98% B and 12.0 min, 2% B. The LC system was equipped with an Atlantis T3 Prep column (250×10 mm, 5 μm particle size, Waters, Ireland), and the T-connection split after the column, which divided the efflux 1:9, directing it to YL9181 ELSD superior sensitivity detector (Young Lin Instruments Co. Ltd., Korea) and Waters fraction collector I (Advantec, Japan) accordingly. The injection volume was 100 μl. The fraction collector was set to collect fractions every three minutes for a total of 12 minutes. Fractions were dried in a Speed-Vac and then resuspended in 100 μl water. The activity of fractions was examined in an induction assay as described above. The assay was performed in a 96-well MatTek plate (100 μl:100 μl).

### Lactate quantification

Cells were grown for 5 h in DMEM in the presence or absence of glucose. CM was collected as previously described. The concentrations of lactate in the CM were quantified using a lactate assay kit (MAK064-1KT, Sigma-Aldrich) according to the manufacturer´s instructions.

### Generation of mutants

Mutations in the genes involved in lactate metabolism were created using fusion PCR. All PCR reactions were performed using high-fidelity Phusion DNA polymerase (Thermo Scientific). Primers used for generation of mutants are listed in Table 1.

First, up- and downstream regions of *ldhA* (ldhA_up_fw:ldhA_up_rev and ldhA_dn_fw:ldhA_dn_rev), *ldhD* (ldhD_up_fw: ldhD_up_rev and ldhD_dn_fw: ldhD_dn_rev), *lldA* (lldA_up_fw:lldA_up_rev and lldA_dn_fw:lldA_dn_rev) and *lctP* (lctP_up_fw:lctP_up_rev and lctP_dn_fw:lctP_dn_rev) were amplified from FAM20 chromosomal DNA using the indicated primer pairs. For the *ldhA* and *lctP* mutants, a tetracycline resistance gene (*tetA*) was amplified from plasmid pACYC184 with primer pairs ldhA_tet_fw:ldhA_tet_rev and lctP_tet_fw:lctP_tet_rev, respectively. For the *ldhD* mutant, a kanamycin cassette was amplified from the pDONR KmR plasmid [55] with primers ldhD_kan_fw and ldhD_kan_rev. For the *lldA* mutant, a spectinomycin cassette [14] was amplified using lldA_spc_fw and lldA_spc_rev. With all acquired PCR products containing overlapping sequences, a fusion PCR reaction was performed in 2 steps. First, a fusion reaction was performed without primers to fuse the upstream and downstream fragments with the resistance cassette. The resulting fusion products were then amplified with the upstream forward primer and the downstream reverse primer for the respective constructs. The constructs were integrated into the genome of *N*. *meningitidis* FAM20 using homologous allelic replacement following spot transformation with purified PCR fragments. The correct location and sequence of the strains were confirmed by sequencing (MWG Eurofins).

### Growth curve analysis

Wild-type FAM20, Δ*lctP*, Δ*ldhA*, Δ*lldA* and Δ*ldhD* mutants were resuspended in DMEM (A1443001, Thermofischer) supplemented with glutamax and either 25 mM glucose, 10 mM L- or D- lactate at an OD_600_ of 0.1 and grown under agitation at 37°C in a 5% CO_2_ environment. The OD_600_ was measured every hour for 10 h.

To determine if lactate exerts bactericidal effect, induction assays were performed as previously described with initial concentration of 10^7^ CFU/ml. The bacteria were grown for 3 h under static conditions. After the 3 h incubation DMEM or DMEM containing L-lactate at a final concentration of 50 mM was added to the suspension at a 1:1 volume ratio and bacterial viability was determined at the end of 8 h.

### ATP and NAD^+^/NADH measurements

Samples were prepared as described above for induction assays. After 3 hours of incubation, prewarmed DMEM, CM or DMEM supplemented with 10 mM L-lactate was added. Samples were taken 10, 20 and 40 minutes after addition and frozen at -80°C. The OD_600_ of the samples was also recorded. The total ATP concentration was measured using a BacTiter-Glo Microbial Cell Viability Assay (Promega) according to the manufacturer’s instructions. Carbonyl cyanide *m*-chlorophenyl hydrazone (CCCP, Sigma) was dissolved in DMEM and used as a positive control at a final concentration of 25 μM. The NAD^+^/NADH ratio was determined with the NAD/NADH-Glo Assay (Promega) according to the manufacturer’s instructions.

### Microcolony dispersal upon oxygen depletion

*N*. *meningitidis* FAM20 was resuspended (10^7^ CFU/ml) in prewarmed medium containing 1% FBS and filtered through a 5-μm pore filter to break apart preexisting bacterial aggregates. The bacteria were incubated in 24-well glass-bottom plates (MatTek) in 1 ml at 37°C in a 95% air/5% CO_2_. At 1.5 h, the oxygen concentration was set to 0%. Oxygen depletion was conducted by a 95% N_2_/5% CO_2_ flow. At 5 h, the oxygen concentration was set to 95% air/5% CO_2_. The bacteria were observed under a live-cell microscope (Axiovert Z1, Zeiss). Three images per well were acquired every 10 min for 8 h using a 40× objective.

### Statistical analysis

Differences between two groups were analyzed using two-tailed and unpaired Student *t*-tests. Differences between multiple groups were analyzed with an analysis of variance (ANOVA) followed by a Bonferroni post-test. For time-lapse microscopy, statistical analysis was used to analyze the differences between the lengths of the dispersal phases. *P*-values below 0.05 were considered statistically significant. The statistical analysis was performed using Microsoft Excel (2011) or GraphPad Prism software, version 5.

## Supporting information

S1 Fig FAM20 adherence and microcolony dispersal on 100% confluent epithelial cell layer. (A) The dispersal of FAM20 microcolonies on FaDu cells at 100% confluence was observed with live-cell time-lapse microscopy. Representative images are shown. Scale bar, 10 μm. (B) Adherence of wild-type FAM20 and its isogenic Δ*pilC1* mutant. FaDu epithelial cells were grown to 100% confluence and infected at MOI of 10 for 4 h. Unbound bacteria were washed away and adhered bacteria were quantified. Data represent the mean ± SD of three independent experiments in duplicates.
(EPS)

S2 Fig Induction of rapid synchronized microcolony dispersal is not FaDu cell-specific. (A) FAM20 infecting the epithelial FaDu, A549, Detroit 562, and Hec1B cell lines at an MOI of 10. (B) CM from uninfected FaDu, Detroit 562, A549, and Hec-1B epithelial cell lines was collected and added to FAM20 microcolonies in liquid. A black horizontal line in panel B represents the 3 h time point of induction. The microcolony dispersal was examined by live-cell microscopy. Data represent the mean ± SD of two independent experiments. ^*^p < 0.05. ns, non-significant.
(EPS)

S3 Fig Treatment with control medium does not influence microcolony dispersal. As a negative control for the cell-conditioned medium treatments in Fig 2A, DMEM was treated with protease inhibitor, proteinase K, trypsin, chymotrypsin, DNase I, RNase A, EDTA, EGTA or 95°C heat prior to addition to the FAM20. A black horizontal line in the panel represents the 3 h time point when treated control medium was added to preformed microcolonies. Data represent the mean ± SD of three independent experiments.
(EPS)

S4 Fig Lactate separates into fraction 2 by reverse HPLC. HPLC fractionation was performed to separate the D-lactate (50 mM) by an acetonitrile and water gradient. Fractions were collected every three minutes for a total of 12 min. Activity of fractions 1, 2, 3 and 4 was examined in an induction assay by addition to FAM20 microcolonies. D-lactate (50 mM) was used as a lactate control. A black horizontal line in the panel represents the 3 h time point when fractions were added to preformed microcolonies. Data represent the mean ± SD of one experiment, a single sample with three images were acquired per well.
(EPS)

S5 Fig Lactate quantification in CM from cells grown in the presence and absence of glucose. Lactate quantification was performed to examine the concentration in CM collected from cells grown in the presence (+) and absence (-) of glucose. Data represent the mean ± SD of lactate concentration from samples collected three times independently in duplicate. ^*^p < 0.05.
(EPS)

S6 Fig Lactate is not involved in *N*. *meningitidis* bacterial lysis. FAM20 were resuspended (10^7^ CFU/ml) in DMEM containing 1% FBS. After 3 h incubation, DMEM or DMEM containing L-lactate at a final concentration of 50 mM was added to the suspensions. The bacteria were further incubated for 5 h. The CFU/ml was measured every hour for total of 8 h. One representative growth curve out of two is shown.
(EPS)

S7 Fig Affect of CCCP on ATP concentration. Samples were taken 10, 20 and 40 min after addition of DMEM or CCCP to FAM20 microcolonies and the concentration of ATP was measured. Data represent the mean ± SD of two independent experiments in duplicates.
(EPS)

S1 Movie Microcolony dispersal can be induced by the addition of cell-conditioned medium (CM). CM from 100% confluent FaDu cells were collected and sterile filtered. The bacteria were first allowed to form microcolonies in DMEM for 3 h. CM was then added to the microcolonies at a 1:1 volume ratio, and the timing of dispersal was examined with live-cell time-lapse microscopy. The time lapse begins 10 min after the addition of the CM and lasts for 20 min.
(MOV)

S2 Movie The addition of DMEM control medium does not induce the dispersal of microcolonies. Bacteria were allowed to form microcolonies in DMEM for 3 h. Non-conditioned DMEM was added as a negative control at a 1:1 volume ratio, and the timing of dispersal was examined with live-cell time-lapse microscopy. The time lapse begins 10 min after the addition of the DMEM and lasts for 20 min
(MOV)
